# Intrinsic Control in Defects Density for Improved ZnO Nanorod-Based UV Sensor Performance

**DOI:** 10.3390/nano10010142

**Published:** 2020-01-13

**Authors:** Abu ul Hassan Sarwar Rana, Shoyebmohamad F. Shaikh, Abdullah M. Al-Enizi, Daniel Adjei Agyeman, Faizan Ghani, In Wook Nah, Areej Shahid

**Affiliations:** 1 Intelligent Mechatronics Engineering/Smart Device Engineering, Sejong University, Seoul 05006, Korea; 2 Department of Chemistry, College of Science, King Saud University, P.O. Box 2455, Riyadh 11451, Saudi Arabia; sshaikh1@ksu.edu.sa; 3 Department of Energy and Materials Engineering, Dongguk University-Seoul, Seoul 04620, Korea; aadaniel@dongguk.edu; 4 Environmental Welfare Research Center, Korea Institute of Science & Technology, Hwarangno 14 gil 5, Seongbuk–gu, Seoul 02792, Korea; t13564@kist.re.kr (F.G.); niw@kist.re.kr (I.W.N.); 5 Department of Electrical Engineering, National University of Sciences and Technology, Islamabad 44000, Pakistan; areejshahid.146@gmail.com

**Keywords:** ZnO nanorods, doping, UV sensor, defects, mobility, responsivity, nanomaterials

## Abstract

Hitherto, most research has primarily focused on improving the UV sensor efficiency via surface treatments and by stimulating the ZnO nanorod (ZNR) surface Schottky barriers. However, to the best of our knowledge, no study has yet probed the intrinsic crystal defect generation and its effects on UV sensor efficiency. In this study, we undertake this task by fabricating an intrinsic defect-prone hydrothermally grown ZNRs (S1), Ga-doped ZNRs (S2), and defect-free microwave-assisted grown ZNRs (S3). The defect states were recognized by studying X-ray diffraction and photoluminescence characteristics. The large number of crystal defects in S1 and S2 had two pronged disadvantages. (1) Most of the UV light was absorbed by the defect traps and the e–h pair generation was compromised. (2) Mobility was directly affected by the carrier–carrier scattering and phonon scattering processes. Hence, the overall UV sensor efficiency was compromised based on the defect-induced mobility-response model. Considering the facts, defect-free S3 exhibited the best UV sensor performance with the highest on/off ratio, the least impulse response time, the highest recombination time, and highest gain-induced responsivity to 368 nm UV light, which was desired of an efficient passive metal oxide-based UV sensor. Our results were compared with the recently published results.

## 1. Introduction

Owing to certain limitations in optoelectronics, such as an inevitable requirement of direct and wide bandgap materials, ZnO has been researched as an alternative to Si technology. With a broad direct bandgap of 3.37 eV and comparatively higher exciton binding energy, which is higher than GaN (60 meV at room temperature), ZnO demonstrated unique optoelectronic characteristics with biocompatibility. Recently, numerous studies have proposed replacing Si optoelectronic technology with ZnO [1,2,3,4]. ZnO has already been fabricated with different nanostructured polymorphic shapes, such as nanorods, nanowires, nanoflowers, nanotubes, nanotetrapods, nanowalls, and nanoparticles [5,6,7,8]. The high surface-to-volume ratio makes these nanostructures an important candidate for optoelectronic devices [9].

ZnO is known for changes in optoelectronic characteristics depending on the crystal defect density. The generation of defects is yet a very controversial topic, however, Oxygen vacancies have been considered as the most dominating donor defect in ZnO crystal lattice. The topic is so pervasive that it leads the researchers to intensively probe into the defect density-oriented changes in optoelectronic characteristics of ZnO [10]. ZnO has been fabricated using multiple methods, such as pulse laser deposition, vapor–liquid–solid, metal organic chemical vapor deposition, molecular beam epitaxy, hydrothermal aqueous chemical growth, and microwave-assisted growth (MAG) methods [11,12,13,14,15,16]. ZnO defect density directly depends on the growth method. Sol–gel methods, including hydrothermal method, are facile, cheap, and user-friendly methods that are in commercial use. However, they generally yield a low crystalline quality nanostructure. Although the other aforementioned methods yield high quality nanostructures, they require sophisticated equipment and involve high costs and temperatures which restrict the use of multiple substrates. We employed the MAG method for ZnO nanorod (ZNR) growth because it is facile, user friendly, commercially benign, and yields the best quality nanostructures in a few minutes.

ZnO is known for multidisciplinary sensor applications because of its strategic location on the borderline of organic and inorganic compounds [17,18]. Most importantly, it has potential applications in photodetectors and UV sensors because of the surface chemisorbed oxygen species and high level absorption in the UV band [19,20]. Photodetector applications require fast response and recovery times, large on/off ratios, and high photo responsivity [21]. ZNRs are the suitable candidates for these requirements owing to their high surface-to-volume ratio. However, because of certain limitations of intrinsic crystal defects in ZnO and their deteriorating effect on UV sensitivity, many researchers have used surface functionalization, polymer coatings, and doping to improve the sensor efficiency [22]. Others have underrated vertical ZNRs and have presented horizontal and bridging ZNR structures to improve the UV sensor efficiency [23,24]. We believe that more studies should focus on improving the defect density-oriented crystal structure to yield the best possible results from intrinsic vertically aligned ZNR-based sensor.

The prime goal of this study is to provide an intrinsic control in crystal defect density, which decreases the majority charge carrier concentration (*N_d_*) in the channel, which in turn reduces the dark current in the device. This also increases the channel mobility (*ρ*), which directly increases the carrier recombination time (*τ*), reduces the impulse response time (*t*), increases the device responsivity (*R*), and thence improves the overall UV sensor efficiency of untreated intrinsic ZNRs. To compare the defect density-oriented UV sensor efficiency, we fabricated three samples—hydrothermally grown ZNRs (S1), Ga-doped ZNRs (S2), and MAG ZNRs (S3). It has been inferred from the structural and optical characteristics that S3 has the best crystalline quality that curtailed the generation of defects. Based on the mobility-response model, S3 demonstrated the best results with the largest on/off ratio, the shortest *t*, and the highest photoconductive gain-induced *R*. Unlike previous studies, we established that n-doping was not an effective method to improve the UV sensor efficiency. This study confirms that an intrinsic control in defect density is an important and adept method to improve the UV sensor performance than using surfactants to stimulate the ZNR surface Schottky barrier or using ZNR polymer coatings to enhance the UV absorption.

## 2. Materials and Methods

### 2.1. Substrate Preparation and Cleaning

For device fabrication, all the chemicals and substrates were purchased from Sigma Aldrich (Seoul, Korea) and used without any further purification. The samples S1, S2, and S3 were fabricated on glass substrates (CAS: 60676-86-0, purity 99%). The substrates were first dry-cleaned with N_2_. They were then rinsed using an ultrasonic bath for 2 min each in acetone (C_3_H_6_O) (CAS: 67-64-1, purity 99%), isopropyl alcohol (C_3_H_8_O) (CAS: 67-63-0, purity 99%), and distilled water. Further, the substrates were annealed at 300 °C and dry-cleaned with N_2_ to remove the remaining impurities on the surface.

### 2.2. Device Fabrication

The first step in device fabrication was the bottom electrode deposition. To make an Ohmic contact with ZnO, silver (Ag) was deposited on the substrate as a bottom electrode. For vertical ZNR fabrication on the substrate, a ZnO buffer layer is inevitable [25]. Hence, the 0.029 M ZnO buffer solution was produced by ultra-sonicating zinc acetate dihydrate (Zn(CH_3_COO)_2_·2H_2_O) (CAS: 5970-45-6, purity 99%) and n-propanol (C_3_H_8_O) (CAS: 67-63-0, purity 99%) for 30 min. The formed solution was then pasted on the substrate’s surface followed by annealing at 300 °C for 20 min.

The next step was the vertical ZNR fabrication. S1 and S2 were fabricated using a convective hydrothermal method while S3 was fabricated by the irradiative MAG method [9,15,16]. Three different solutions were prepared for S1, S2, and S3 during this process. For S1 and S3, the homogeneous growth solutions were prepared by mixing equimolar (25 mM) zinc nitrate hexahydrate (Zn(NO_3_)_2_·6H_2_O) (CAS: 10196-18-6, purity 98%) and methenamine (C_6_H_12_N_4_) (CAS: 100-97-0, purity 99%) in distilled water. For S2, 1.315 mM gallium nitrate hydrate (Ga(NO_3_)_3_·xH_2_O) (CAS: 69365-72-6, purity 99%) was added and stirred into the abovementioned solution to fix the Ga-doping content to 2%. The doping % was measured using the formula: *Ga%* = *M_Ga_*/(*M_Ga_* + *M_Zn_*) × *100%,* where *M_Ga_* and *M_Zn_* are the Ga and Zn molar concentrations, respectively. To make the saturated solution, the chemicals in three different containers were stirred continuously for 1 h.

**Figure 1:**
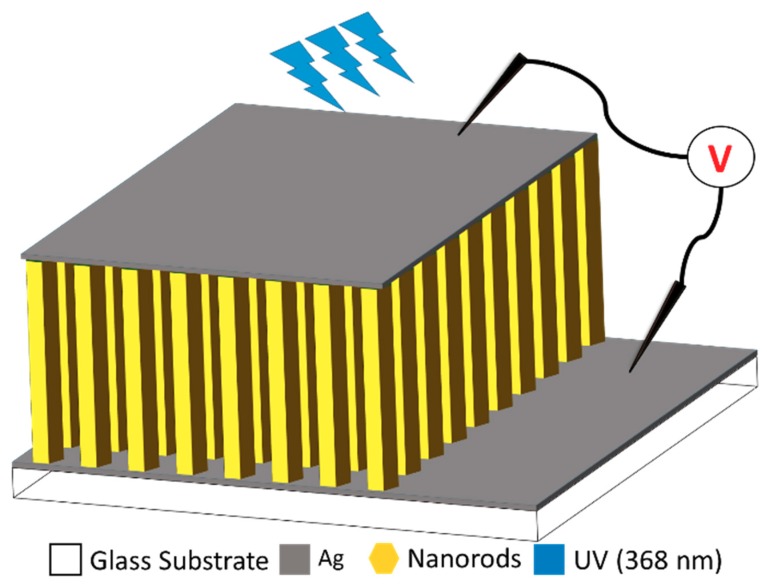
Schematic representation of the fabricated devices.

Afterwards, the three different glass substrates with bottom electrodes and a ZnO nucleation layer were immersed into their corresponding solutions for the ZNR growth. For S1 and S2, the autoclaves were placed on hotplates for 6 h at 90 °C with magnetic stirring at the bottom. The samples were then removed from the autoclaves and cleaned with distilled water. On the contrary, the S3 solution was placed in an 850 W domestic microwave oven and subjected to 2.45 GHz microwave radiations for 20 min. The ZNRs were grown using a pulsed microwave heating process [16]. After 20 min, the sample was then removed from the solution and cleaned with distilled water. Finally, the top Ag electrode was deposited on the samples to make them metal-semiconductor-metal (MSM) devices. Schematic Figure 1 illustrates the device structure and fabrication layout. The active 1 μm thick ZNR layer is sandwiched between the top and the bottom Ag electrodes providing an Ohmic contact to the MSM device on glass substrate. The electrical connections are provided to study the sensor response under the dark and illuminated conditions. Upon 368 nm UV illumination, the e–h pairs are generated in the active ZNR layer and the provided potential difference drives them towards the electrodes and the rise or fall of current is recorded accordingly.

### 2.3. Characterization Tools

This section explains the type of equipment used to characterize the UV sensor devices with their particular characteristic details. Scanning electron microscope (SEM: Hitachi S4700, Hitachi, Tokyo, Japan) images were used to examine the morphology and measure the physical dimensions of the ZNRs. X-ray diffraction (XRD: Rigaku D/MAX–2500 V/PC, Tokyo, Japan) and photoluminescence (PL: FLS 1000, Livingston, UK) spectroscopies were utilized to examine the crystalline structure and the optical characteristics of the devices, respectively. The defect states were also analyzed and inferred from the XRD and PL results. The elemental characteristics, including the weight and atomic percentages of individual elements in a compound, were confirmed by energy dispersive X-ray microanalysis (EDXMA) attached with the SEM equipment (Hitachi S4700, Hitachi, Tokyo, Japan). The composition and oxidation states of the compounds were tested with X-ray photoelectron spectroscopy (XPS: PHI 5000, Versa Probe Ul—vac PHI with Al Ka radiation Monochromator 1486.6 eV, Kanagawa, Japan), Hall–effect characterization was used to measure the real-time carrier concentrations of the samples. Electrical characteristics and photonic response were measured with Keithley 4200A-SCS parameter analyzer (Keithley Instruments, Solon, OH, USA). The current–voltage (*I–V*) characteristics were measured by sweeping the voltage from −2 to 2 V and the photonic impulse response was measured at the constant voltage of 2 V for 368 nm UV on/off conditions.

## 3. Results and Discussion

### 3.1. ZnO Nanorods Morphology and Physical Characteristics

**Figure 2:**
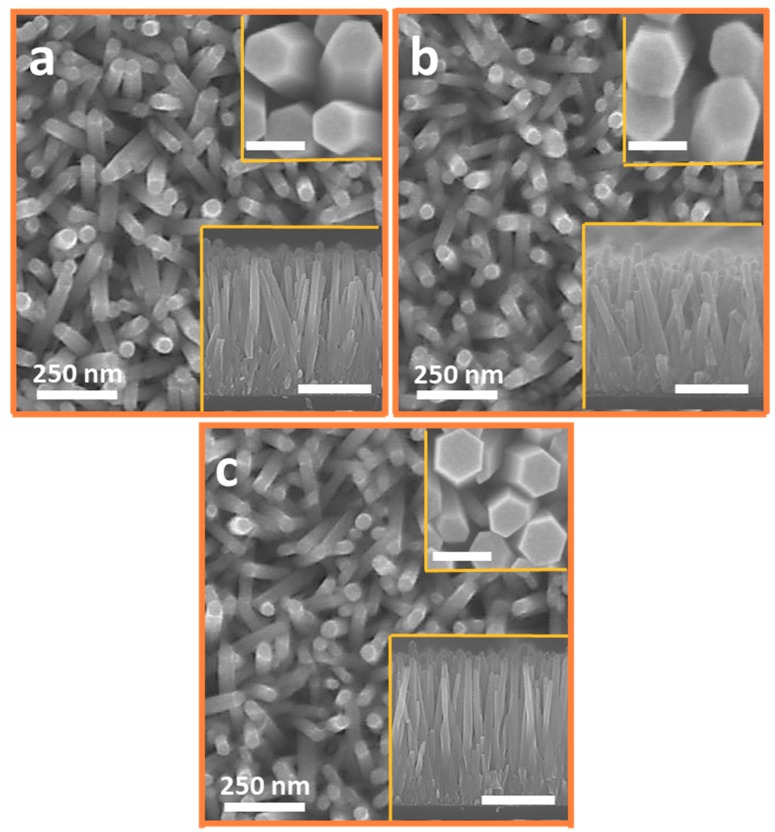
Plain view SEM images of (**a**) hydrothermally grown ZnO nanorods (ZNRs) (S1); (**b**) Ga-doped ZNRs (S2); and (**c**) defect-free microwave-assisted grown ZNRs (S3). The top and the bottom insets are the corresponding magnified images and cross-sectional images of the same ZNRs, respectively. The scale bar in the top insets is 100 nm and in the bottom insets 500 nm.

Figure 2 shows the plain view ZNRs morphology imaged using SEM. It is evident that all the ZNRs are well grown perpendicular to the substrate. The point to ponder is that the ZNR dimensions were intentionally synchronized to effectively study the real essence of change in UV sensor characteristics because of crystal defect states-induced changes in mobility than in the surface-to-volume ratio. For S1 and S2, the controlled hydrothermal temperatures and growth times aided in synchronizing the surface morphologies and aspect ratios. However, for S3, where the microwave intensity is not liable to be controlled in a domestic oven, pulsed microwave heating was used to synchronize S3 morphology and aspect ratio with S1 and S2 [16]. One microwave exposure to a 25 mM solution yielded 250 nm (±5 nm) long heterogeneously nucleated ZNRs. Hence, the solution was replaced four-times to achieve the total length of 1 µm (±20 nm), as shown in the bottom insets of Figure 2. The ZNRs’ diameter remained constant for all the four rounds of replaced solution because of the presence and the development of very stable ZNR lateral walls that allowed axial ZNR growth. The ZNRs’ diameter was synchronized at approximately 70–90 nm for all the samples. The Figure 2 top insets show the high magnification SEM images of ZNRs with a scale bar of 100 nm for reference and the bottom insets show the cross-sectional SEM images with the scale bar of 500 nm for reference.

### 3.2. XRD Structural and PL Optical Characteristics

**Figure 3:**
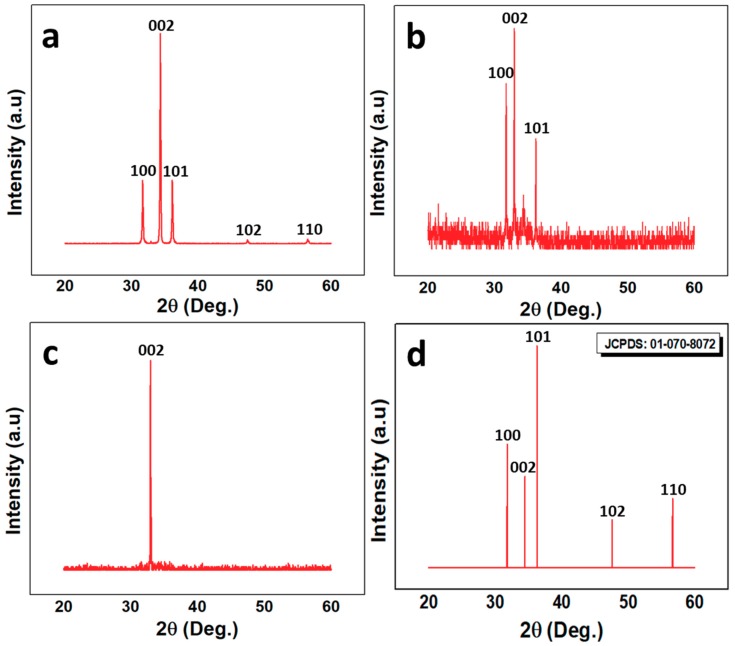
XRD analysis of (**a**) S1; (**b**) S2; (**c**) S3; and (**d**) JCPDS reference for ZnO.

The XRD and PL results reflect the ZnO defect density and crystal structure. Figure 3a–c reports the XRD structural characteristics of the three samples used for the experiment. It is evident that all the samples have a hexagonal wurtzite phase and are void of any peak related to any element except ZnO. All the peaks matched well with the JCPDS card number 01-070-8072, which is shown in Figure 3d. The highest peak along [002] confirms the vertical alignment of the ZNRs, which is in line with the SEM images. The multiple peaks in S1 and S2 show that both samples were polycrystalline. However, a single peak along [002] confirms the S3 single crystal structure and perfect alignment along the [002] direction. The crystalline quality was further compared based on the full width at half maximum (FWHM) and the crystallite size of ZNRs. The crystallite/grain size was measured using Scherrer formula [26,27]. The lattice constant (*C*) was measured by the formula:

(1) $$C=\frac{\lambda }{sin\theta }.$$

In Equation (1), *λ* is the X-ray wavelength and *θ* is the angle corresponding the 002 peak. The strain (*ε*) in the ZNRs was calculated by the relation:

(2) $$\varepsilon =\frac{C-C_{o}}{C_{o}}\times 100,$$

where *C* is the lattice constant of the individual samples and *C_o_* is the lattice constant of the ideal strain-free ZnO, which is 5.20 Å. The details of the structural characteristics are listed in Table 1. The best crystalline quality was shown by S3 with the least FWHM and the highest crystallite size. Hence, it could be inferred that S3 had the least number of defects and the best crystalline structure, which helped to improve the UV sensor efficiency of S3 as the defects relationship with the sensor efficiency is discussed in detail in Section 3.4.

**Figure 4:**
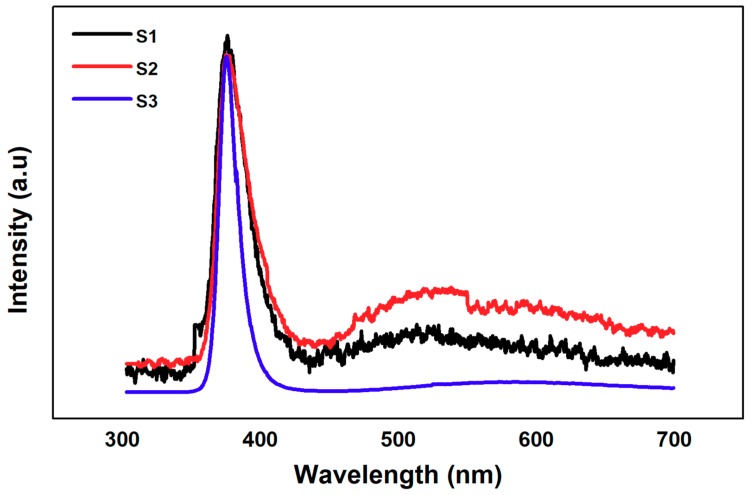
Photoluminescence (PL) optical characteristics and defect analysis of S1, S2, and S3.

Figure 4 shows the PL optical characteristics of the samples. All the samples showed a high intensity peak in the UV region because of the exciton recombination process and a low intensity broad peak in the visible region because of the defects. Notwithstanding, the overall behavior of three graphs are similar and the peak intensities are different in the visible region, which indicate a change in the crystalline structure of the three samples. The UV-to-visible band peak ratio is an important factor to analyze the defect centers and the crystalline quality of the ZNRs. The highest ratio is exhibited by S3 with the least peak intensity in the visible region owing to minimum defects. Hence, the XRD structural and the PL optical characteristics confirmed that S3 had the best crystalline quality with the least number of crystalline defects.

### 3.3. EDXMA and XPS Analyses

**Figure 5:**
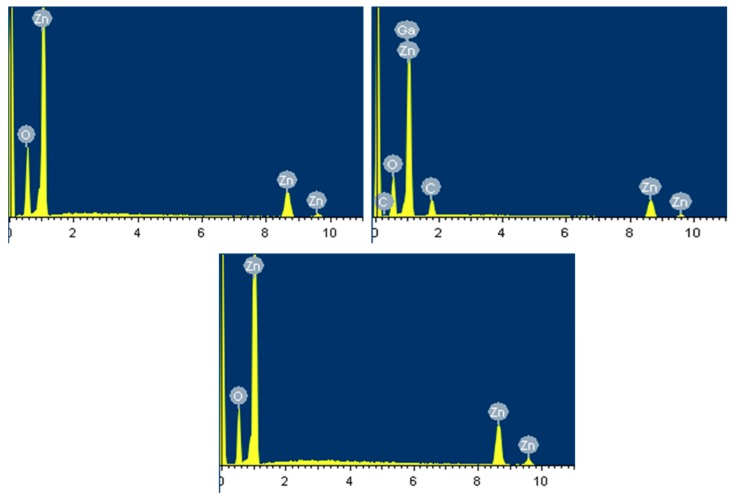
Energy dispersive X-ray microanalysis (EDXMA) of (**a**) S1; (**b**) S2; and (**c**) S3.

The EDXMA results were used to confirm the elemental characteristic compositions in the fabricated compounds along with their individual atomic and weight percentage measurements. It was also used to confirm the Ga incorporation into the ZnO crystal lattice via doping. Figure 5 shows the EDXMA results for S1, S2, and S3, which was measured in KeV on x-axis against intensity on y-axis. The percentage weight and atomic composition of individual elements are reported in Table 2. The large peak at 0.1 KeV is the strobed electronic peak because of noise, which are present in all the samples and liable to be neglected. It was confirmed that the major elements in the sample compounds were zinc and oxygen. The large Ga peak at approximately 1 KeV in Figure 5b—with weight and atomic % of 2.18 and 2.64, respectively—confirms the successful Ga incorporation into the ZnO crystal lattice by doping. The small C kinks in S2 are the impurities incorporated during growth. However, no foreign element was found in intrinsic S1 and S3. It is evident that S1 (Figure 5a) and S2 (Figure 5b) are high in Zn and low in O atomic ratios. It is because of the presence of Oxygen vacancies in the ZnO crystal lattice, which are primary ZnO defects. However, EDXMA for S3 (Figure 5c) shows a meager difference in Zn to O atomic ratios because of the immutable nature of S3 with least defects. The EDXMA results were in accordance to the XRD and PL results reported in Figure 3 and Figure 4, respectively.

**Figure 6:**
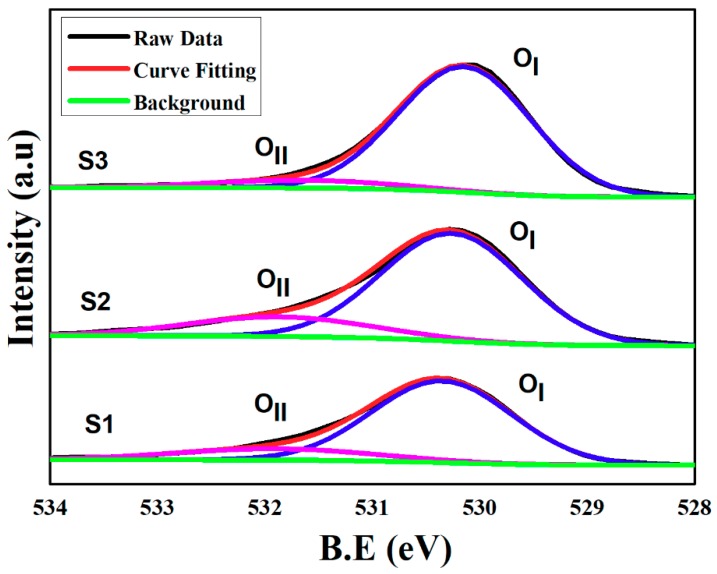
XPS analysis of S1, S2, and S3.

The composition and oxidation states of ZnO were characterized with XPS and the results are shown in Figure 6. The deconvolution of O1s spectrum indicates that all the samples consist of the Oxygen metal bond O_I_ formed at 530.35, 530.30, and 530.34 eV and the Oxygen deficiency region O_II_ at 531.89 eV, 531.91 eV, and 531.85 eV, respectively. The presence of O_II_ confirmed that the Oxygen vacancies are the dominant parameter to generate ZnO intrinsic crystal defects [28]. Moreover, S1 shows the 16.23% of Oxygen vacancy defects lower than that of S2 with 19.02% Oxygen vacancies. On the contrary, S3 depicts a controlled behavior with only 7.88% of Oxygen vacancy defects. The analysis confirmed the very strong Zn–O bond and the crystal defects are intrinsically controlled by the method of choice used for ZnO crystal growth. Moreover, the O_I_ sweep towards lower binding energy indicates that the vacancy point defects are depressed in the crystal lattice of S3. Hence, ZnO with intrinsically controlled defects was confirmed by the XPS analysis.

### 3.4. Proposed UV Sensor Mechanism

We propose a better UV sensor mechanism without using any surfactants, such as polymers, to stimulate the Schottky barrier at the ZNR surface. Hence, the ZnO intrinsic properties are to be considered for better sensor efficiency. It is already established that quantum efficiency (*η*) is directly proportional to responsivity (*R*):

(3) $$R=\eta \frac{\lambda \left(\mu m\right)}{1.24},$$

where *λ* is the UV source wavelength in microns. One of the relations for *η* is

(4) $$\eta =\left(1-r\right)\left(1-e^{-\alpha d}\right)\zeta ,$$

where *α* is the absorption coefficient, *r* in the reflectivity, *d* is the film thickness of the photodetector, and *ζ* is the fractional number of photons absorbed by the photodetector. The factors *α* and *r* are the characteristic constant to the material at particular thickness, and are antithetical to each other. The values strictly depend upon the ZNR active layer film thickness “*d*”. Because the *d* was synchronized to 1 µm for all three samples (S1, S2, and S3), the “*r*” and “*α*” were not considered to alter the UV sensor efficiency. On the contrary, it is “*ζ*” because of which the UV sensor efficiency significantly varies. This is the reason why only “*ζ*” was considered to improve the UV sensor efficiency rather than “*r*” and “*α*”. Hence, *η* could be maximized by maximizing *ζ*. All the incident photons may not generate e–h pairs. Some of them may generate phonons because of dielectrics and some recombine shortly because of defects. Hence, the factor *αd* is constant for all the fabricated samples (S1, S2, and S3) and *ζ* could be maximized by a very careful fabrication of the photodetector material.

**Figure 7:**
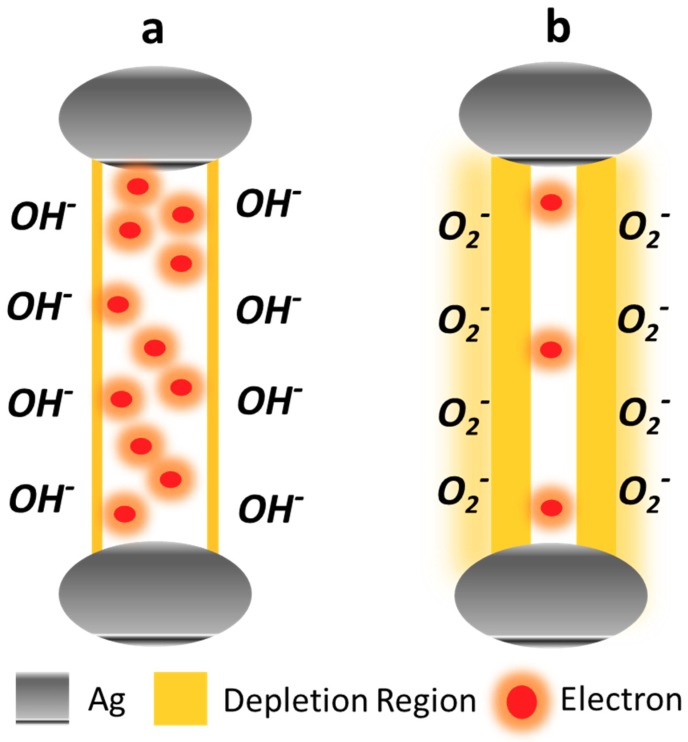
Channel layer e distribution and surface Schottky barrier in (**a**) defect-prone S1 and S2 with OH– surface accumulation layer and (**b**) defect-free S3 with chemisorbed surface oxygen.

The ZnO defect generation process and their degenerative role in demeaning the ZnO optoelectronics performance has already been discussed in previous studies [10]. For S1 and S2, the hydrothermal growth conditions provided a feeble lattice energy to the atoms to grow atop the proper wurtzite ZnO crystal planes and aided in intrinsic defect generation in terms of Oxygen vacancies. The performance was further deteriorated by the formation of OH^–^ accumulation layer on the ZNR surface because of the samples’ immersion into the low temperature hydrothermal solution for hours. The ambient H reacts with the chemisorbed O^2–^ at the ZNR interface and formed the very stable accumulation layer [29,30,31], as demonstrated in Figure 7a. It also released the chemisorbed e from ZNR surface into the channel, which affects the ZNR *ρ*, where the overall factor *ζ* was compromised. On the contrary, the thermal energy transferred via high power microwaves to the atoms in S3 sufficed to grow atop the appropriate crystallographic planes in the lattice. The process reduced the defects generation probability for Oxygen vacancies, which are the primary defects in ZnO. As shown in Figure 7b, the ZNR surface Schottky barrier increased by chemisorption and the channel *ρ* improved because of the channel e transfer to the ZNR surface. The minimum defects generation and accumulation layer removal from the ZNR surface would maximize *ζ* which in turn maximizes *η* and ultimately *R*. This is the proposed UV sensor mechanism for this study. Hence, the objective is to generate an intrinsic ZnO film with minimum defects to maximize the intrinsic *ρ* and *R*.

### 3.5. Current–Voltage (I–V) Characteristics

We investigated the UV on/off ratio by studying the *I–V* characteristics of the devices. The maximum wavelength used for the e–h pair generation could be found with the relation *λ_max_* ≤ *hc*/*E_g_* ≈ 368, where *λ_max_* is the maximum wavelength, *h* is the plank’s constant, *c* is the speed of light, and *E_g_* is the ZnO bandgap energy. The current in the external circuit after the photon illumination is given by Remo’s Theorem. The total current density (*J*) is given by the relation

**Figure 8:**
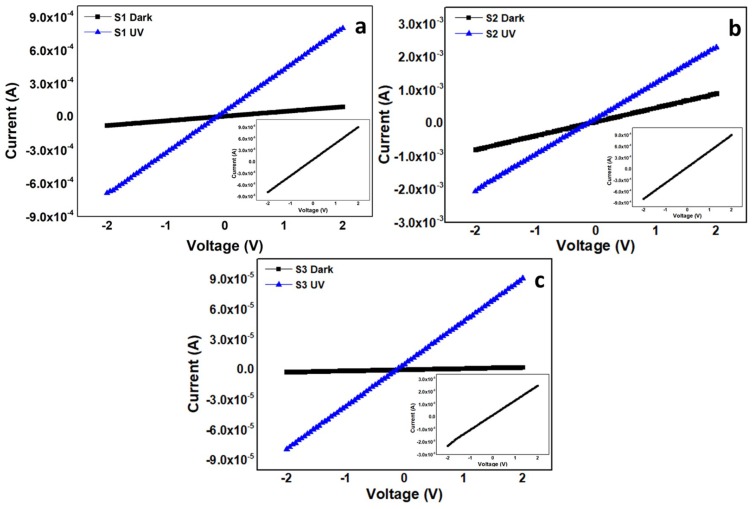
Current–voltage (*I–V*) characteristics of (**a**) S1; (**b**) S2; and (**c**) S3. The insets show the magnified dark current characteristics of the corresponding samples for clarity.

*J* = *J_e_* + *J_h_*, (5)

where *J_e_* and *J_h_* are the electronic and hole current densities, respectively. ZnO is predominantly an n-type material because of the intrinsic donor defects [32,33,34]. Hence, S1 and S3 showed an intrinsic behavior to the external bias. Figure 8 shows the *I–V* characteristics of the samples to dark and 368 nm UV illuminated conditions. The insets show the detailed dark current characteristics. The graphs confirm the formation of a perfect Ohmic contact between ZnO and Ag. The dark current, which is termed as noise in photodetectors, was high in S1 because of the presence of a plethora of donor defects. Similarly, the highest dark current was recorded from doped S2 because of the increase in the doping-induced carrier concentration, as shown in Figure 8b. Nevertheless, studies have reported UV sensitivity improvement via Ga-doping to ZnO [35,36,37], we found that the dark current was comparatively high in S2, which reduced the on/off ratio and degraded its UV sensor performance. Furthermore, the overall ρ was also compromised in S2 because of high carrier concentration-induced increased scattering. Hence, n-doping is not an effective method to improve the UV sensor efficiency. The high dark current intensities in S1 and S2 reduced the on/off ratios to 9 and 2.5, respectively. However, the best results were shown by S3 with the highest on/off ratio of 36 because of the least dark current intensity. The measured carrier concentrations were 1.10 × 10^17^, 1.02 × 10^18^, and 8.33 × 10^13^ for S1, S2, and S3, respectively. Furthermore, S3 showed the highest hall effect mobility value of 2.7 × 10^01^ compared to 1.11 × 10^00^ in S1 and 1.86 × 10^−01^ in S2, which led us to compare UV sensor performance using the mobility response model.

### 3.6. Impulse Response as a Function of Time

The relation for transit time or impulse response (*t*) is

(6) $$t=\frac{W}{V_{e}},$$

where *W* is the device active area and *V_e_* is the electron drift velocity. For a very small impulse response, *V_e_* should be maximum and *W* should be small. Here, the area for all the sensors was synchronized to 1 × 0.5 cm^2^. Hence, the impulse response could only be improved by increasing *V_e_*. The relation between *V_e_* and *ρ* is

(7) $$V_{e}=\rho \cdot E,$$

where *ρ* is the channel electron mobility and *E* is the applied electric field. The factor *E* was constant for all the samples. Hence, *V_e_* is directly related to *ρ*, which is important for better sensor response. Furthermore, the value of *E* should be sufficient to reach the maximum saturated *V_e_*. Hence, it is recommended to use 5–10 V as a constant voltage supply rather than 2 V. However, we used 2 V for pragmatic applications of the devices in low power operations. Hence, *t* and efficiency were tested on a small constant external voltage of only 2 V. High external voltage aids in parting the generated e–h pairs effectively and improves their *V_e_* for better efficiency. However, to test the effectiveness of the fabricated device and the sensor efficiency dependence on the intrinsic crystal structure, we tested the device with mere 2 V, where the generated e–h pair recombination probability was high. 

**Figure 9:**
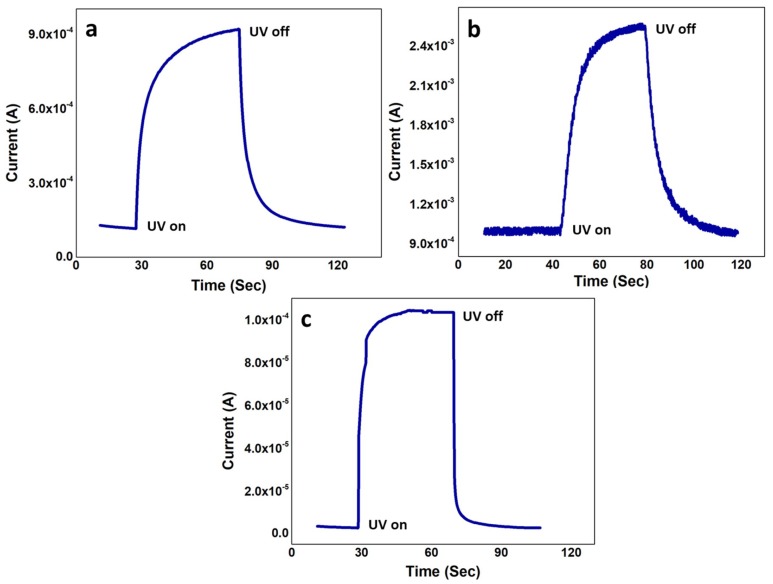
Transient current/impulse response (t) of (**a**) S1; (**b**) S2; and (**c**) S3.

Figure 9 shows the *t* of the three samples for the UV on/off conditions. The velocity saturation was short in S1 and S2 because of increased collision and scattering process and reduced *ρ*, which increased the *t* to 23 s and 29 s, respectively. In S1, the current started increasing at ~27 s and reached its maximum at ~50 s where it saturated because of the e–h pair generation and recombination equality. Similarly, for S2, the current started responding the UV light at ~41 s and saturated at ~70 s. Although *t* for our S1 and S2 are already better considering the vertical passive MSM devices, S3 showed the best impulse response of 9 s, which is extraordinary considering the intrinsic passive MSM UV sensor. The shortest *t* in S3 is because of the best *ρ* induced highest *V_e_*. The least number of crystal defects and immutable crystal structure of S3, as evident in Section 3.2 and Section 3.3, offered minimum resistance to the freely flowing UV generated e in the channel. Not only the carrier–carrier scattering phenomenon was the least, but also the perfect lattice arrangements minimized the phonon–carrier scattering in the S3 channel [38,39]. Overall, the least channel scattering gave the best *ρ*, the direct relationship between *ρ* and *V_e_* gave best *V_e_*, and the inverse relation between *V_e_* and *t* gave least *t* in S3. The whole process gave a sharp rise in current for a very small *t*, as shown in Figure 9c. On the contrary, although the total current intensity would be increased because of the increased e, S2 had longer *t* because of the low mobility-induced decreased *V_e_*. The recorded recovery times for S1, S2, and S3 were 25, 30, and 9 s, respectively. The dark and illuminated current intensities in Figure 9 were synchronized with the *I–V* characteristics in Figure 8.

### 3.7. Quantum Efficiency, Responsivity, and Photoconductive Gain

**Figure 10:**
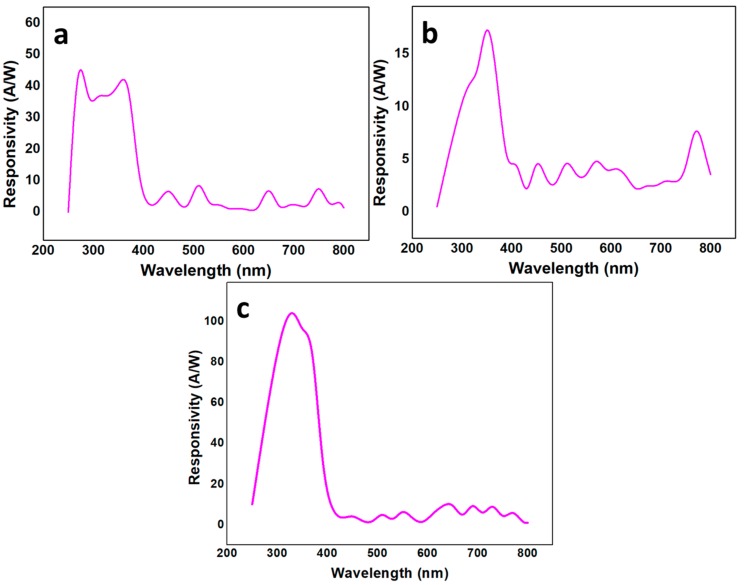
Responsivity as a function of wavelength for (**a**) S1; (**b**) S2; and (**c**) S3.

The *η* could be decreased by the generation of hot carriers, which generate phonon vibrations and facilitate short term e–h pair recombination and absorption near the surface. This in turn facilitates the e–h pair recombination by the presence of dangling bonds. The hot carrier generation was not possible in our case because we operated the device on 2 V. Hence, the near surface absorption because of surface defects should be considered. The graphs in Figure 10 show the *R* of the samples as a function of wavelength. The best *R* was exhibited by S3 operating the device on a mere 2 V, which is better than the recently published reports [40,41,42]. Herein, S3 shows the best *R* of 104 (±1) A/W compared to 44 (±2) A/W and 16 (±4) A/W in S1 and S2, respectively. Hence, the *R* for S3 is 2.3 times larger than *R* for S1 and 6.5 times larger than *R* for S2. Notwithstanding, S3 had the least defects that minimized the UV light absorption by defect traps. Hence, most of the absorbed light was used to generate the e–h pairs, which improved the UV sensor efficiency. Contrarily, the e–h pair generation was minimized because of the UV light absorption by the defect traps in S1 and S2. The *ρ* was also compromised a lot because of the carrier–carrier scattering and phonon scattering processes. The defects also facilitated the e–h pair recombination shortly after their generation. The defects directly affect the carrier recombination time (*τ*), which was maximum in S3 because of the highest *ρ*. The avalanche process through impact ionization further compromised the S1 and S2 performance. However, the avalanche process improved the S3 performance by increasing the on/off ratio of the device, which is evident in the *I–V* characteristics (as shown in Figure 8). Considering the aforementioned facts, S3 showed the largest on/off ratio, the least *t*, and the best *R*, which is desired of an efficient passive MSM UV sensor.

The photoconductive gain (*G*) is inversely proportional to transit time (*t)* and directly proportional to carrier recombination time (*τ)*.

(8) $$G=\frac{\tau }{t}.$$

According to the following relation,

(9) $$G=R\cdot \frac{1.24}{\lambda }\cdot \frac{1}{\eta },$$

where *G* and *R* are directly proportional to each other, and λ is the UV source wavelength in microns. *G* can be calculated by setting *η* = 1 for simplicity. S3 produced highest *G* because of the highest *ρ*, the least *t*, and the maximum *τ*. We believe that many electrons reach the destination in a short time because of the increased *ρ* and many electrons are released by the electrode to maintain the charge neutrality in the circuit. The avalanche process provided further impetus for high *G* in S3 compared to S1 and S2. Hence, the plethora of e collected at the electrode against a single h is the reason for high *G* in S3. Contrarily, this was not true for S1 and S2 because of the high scattering and the low *ρ*. Consequently, the highest *G* yielded the best *R* value for S3. *R*-centric UV-to-visible rejection ratio was also the highest in S3 compared to S1 and S2.

## 4. Conclusions

To epitomize, we studied the intrinsic control in defect density to improve the vertical ZNR-based passive MSM UV sensor efficiency. Three samples each of hydrothermally grown intrinsic (S1), Ga-doped (S2), and microwave-assisted grown (S3) were considered for comparison to support our primary focus. The defect density was confirmed by studying the XRD structural and the PL optical characteristics. It was noted that S3 had the best crystalline structure with the minimum defects. Based on the defect structure, the samples’ carrier concentration-centric mobility was calculated and a mechanism for mobility-based UV sensor performance enhancement was drawn. Based on the experimentation and results, it was postulated that S3 demonstrated the best UV sensor results with the least transit response and recovery times, the highest recombination rate, the highest responsivity, and the best gain. It was also determined that Ga-doping or n-doping was not an effective technique to improve the UV sensor performance. Further, as opposed to using the surface treatments to stimulate the Schottky barrier at the ZNR surface or coating the sensor film with polymers to improve the UV absorption, controlling the intrinsic defect density is recommended to improve the ZnO UV sensor performance. The fabricated sensors have applications in optical biopsy, wearable electronics, defense and space applications, and optoelectronics.

## Figures and Tables

**Table 1:** Structural parameters of S1, S2, and S3.

Sample	2*θ* (Degree)	FWHM (Radian)	*D* (nm)	*C* (Å)	*ε*
S1	34.63 (±0.04)	0.0046 (±0.0002)	30.93	5.284	1.62
S2	34.80 (±0.08)	0.0054 (±0.0004)	24.07	5.299	2.9
S3	34.48 (±0.01)	0.0021 (±0.0002)	67.16	5.204	0.25

**Table 2:** EDXMA weight and atomic compositions of individual elements in S1, S2, and S3.

Element	Weight %	Atomic %
**S1**		
O	27.10 (±0.92)	31.68
C	4.01 (±2.06)	11.84
Zn	66.71 (±0.29)	53.84
Ga	2.18 (±1.42)	2.64
Total	100.00	100.00
**S2**		
O	19.93 (±0.81)	38.87
Zn	80.07 (±0.36)	61.13
Total	100.00	100.00
**S3**		
O	24.47 (±0.20)	48.56
Zn	75.53 (±0.16)	51.44
Total	100.00	100.00
